# Denture Marking for Forensic Identification Using Laser-Marked Stainless Steel Quick Response (QR) Code

**DOI:** 10.7759/cureus.22431

**Published:** 2022-02-21

**Authors:** Shreya Colvenkar, Ravindra SV

**Affiliations:** 1 Prosthodontics, M.N. Raju (MNR) Dental College and Hospital, Sangareddy, IND; 2 Oral Medicine and Radiology, M.N. Raju (MNR) Dental College and Hospital, Sangareddy, IND

**Keywords:** stainless steel, qr code, laser, forensic, denture

## Abstract

Denture marking is an accurate and rapid method to identify unknown individuals in social and forensic scenarios. Numerous published works have attempted to present various techniques for denture marking; however, not all satisfy requirements either in terms of cost, storage of a large amount of information, and their capability to withstand high temperature. Hence, this article describes a simple, cheap, and feasible denture marking system using laser-marked stainless steel quick response (QR) code. A QR code was generated in the dental office, laser marked on stainless steel band, and inserted into a mandibular denture. The code was then read using an app downloaded into a mobile phone giving access to details about the patient. The code was created to store, modify, and view large amounts of information with smartphones, thus allowing quick identification.

## Introduction

The role of denture marking in social and forensic scenarios has long been acknowledged by dentists and forensic odontologists. It helps in identifying dentures and persons in geriatric institutions or postmortem during wars, crimes, and natural and mass disasters. During mass disasters or genocide crimes, quick identification of the deceased becomes a difficult task as the bodies are mostly mutilated beyond recognition. Dental identification has helped in many instances when other methods have failed. Quick identification is usually possible when an identification code is embedded in the dentures. Replacing lost dentures will cause a lot of inconvenience, as it involves getting used to the new fit and appearance. Denture replacement also involves cost, arranging for a dental visit, time, and transportation.

In the literature, denture marking techniques are broadly divided into surface marking and inclusion methods. Surface markers [1,2] are economical but can be easily removed by denture cleansers, abrasives, or antiseptic mouthwash. Surface inclusion techniques consist of the incorporation of a marker, which includes metallic or nonmetallic materials [3], microchips [4], radiofrequency identification tags [5,6], microSD card [7], barcodes [8-10], and lenticular card [11], which vary widely in relation to the inclusion technique and reading.

Inclusion techniques using metallic and nonmetallic labels carry very little information even though they are cheap and easily available. Microchips, barcodes, and radiofrequency identification tags permit rapid identification through the storage of a large amount of information, but these techniques are costly and need sophisticated equipment to read the information. In addition, microchip data could be inscribed only by the manufacturer and not by the dentist. In the lenticular method, the information can never be changed once inserted.

The use of quick response (QR) codes as a method of personal identification in dentures presents most of the requirements recommended in the literature, except for the resistance to high temperatures. QR coding of dentures is simple and cheap, with the potential of storing a large amount of information, thus allowing fast and reliable identification except for the resistance to high temperature [9]. The stainless steel Swedish ID-Band (SDI AB, Sweden) [12], which has become the international standard and FDI World Dental Federation-accepted denture marking system, can withstand temperatures up to 1,100°C but cannot store a large amount of information. This article describes an innovative technique wherein a laser is used to mark QR code on stainless steel band, which is then inserted in the denture, thus allowing storage of huge information together with its resistance to high temperature.

## Technical report

**Figure 1:**
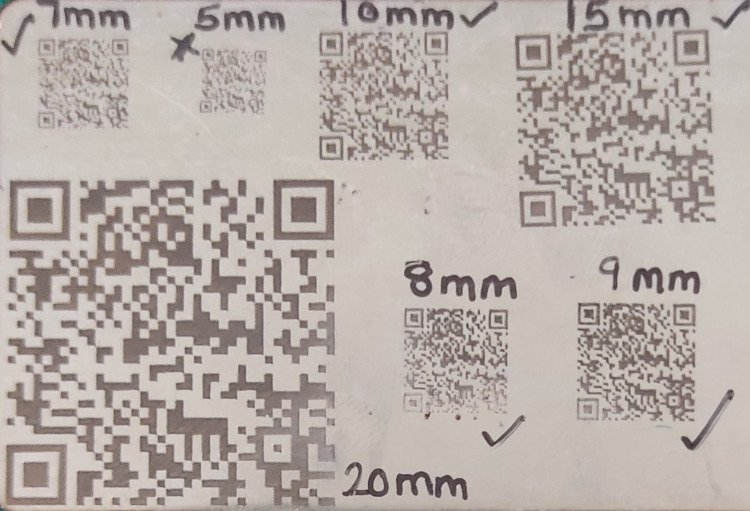
Different sizes of QR codes. QR, quick response.

Generate a QR code having patient details such as name, age, gender, address, medical history, and Aadhar number with a code generator app (Barcode Generator, Kowloon, Hong Kong). Mark the code with the help of a laser machine (KFD-10, Dongguan Kite Laser Technology Co., Ltd, Guangdong, China) on a stainless steel sheet of 0.5 mm thickness. Mark the code in varying sizes of 6 mm, 7 mm, 8 mm, 10 mm, and 15 mm to confirm which size of the code can be decoded with the decoder-enabled mobile camera (Figure 1).

**Figure 2:**
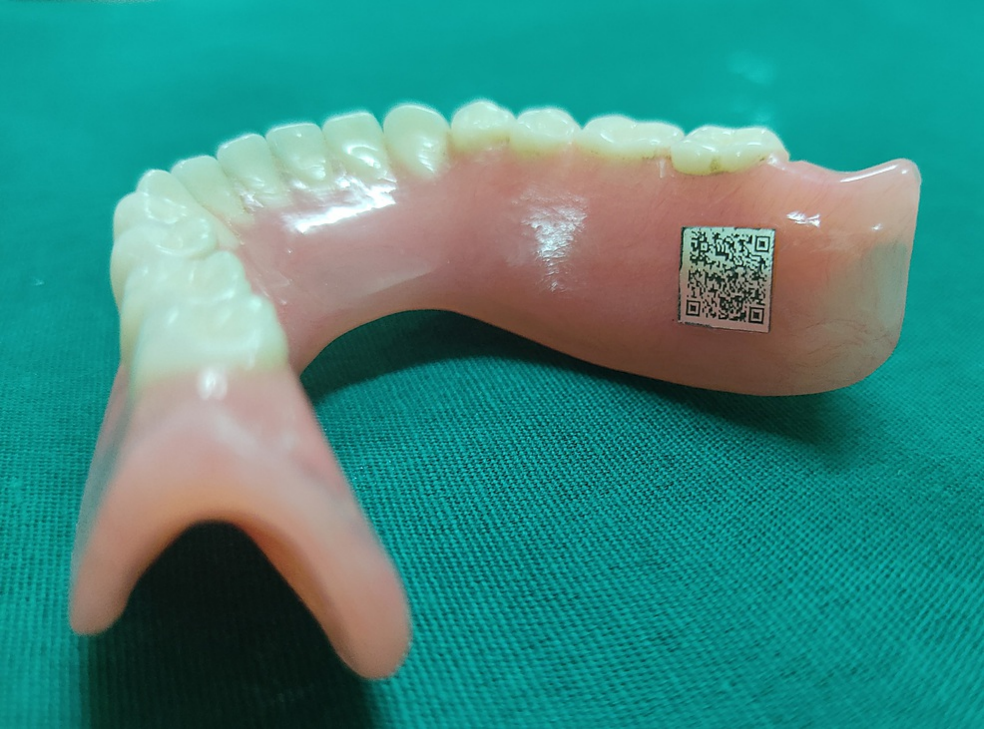
QR code inserted in the denture. QR, quick response.

The 7 mm size could be decoded with ease. Cut the code to a size of 7.2 x 7.2 mm. Process the dentures according to the manufacturer's instructions. Start the incorporation process after disinfecting, cleaning, and drying the prosthesis. Cut a 0.6 mm depression slightly wider than the size of the identifier on the external posterior lingual flange of the mandibular denture using a carbide bur (Zhangjiagang Saimeng Tools Co., Ltd., Jiangsu, China). Incorporate the identifier in the channel and fill the recess with clear, auto-polymerizing acrylic resin (Rapid Repair, Dentsply Int'l, York, PA). In a pressurized container, process the denture (Confident Dental Equipments Private Limited, Bangalore, India) with warm water (1008F, 20 psi) for 15 to 20 minutes. Remove excess acrylic resin and finish and polish the denture to complete the procedure (Figure 2).

**Figure 3:**
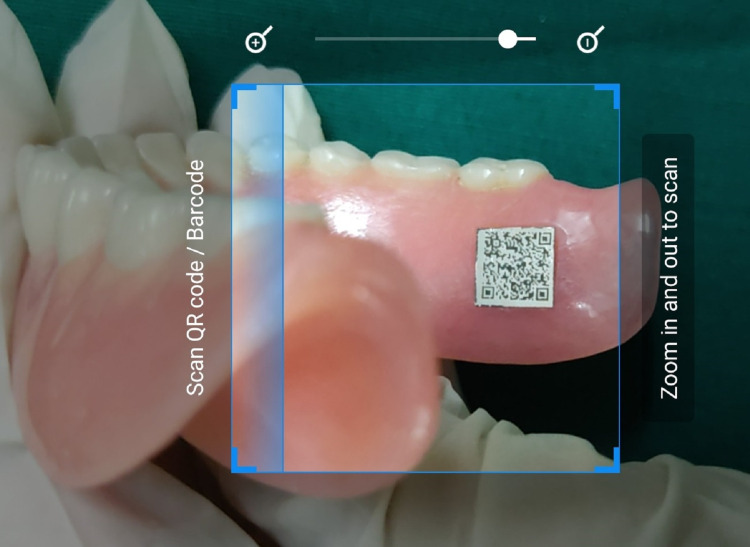
Scanning of the QR code with a mobile phone. QR, quick response.

**Figure 4:**
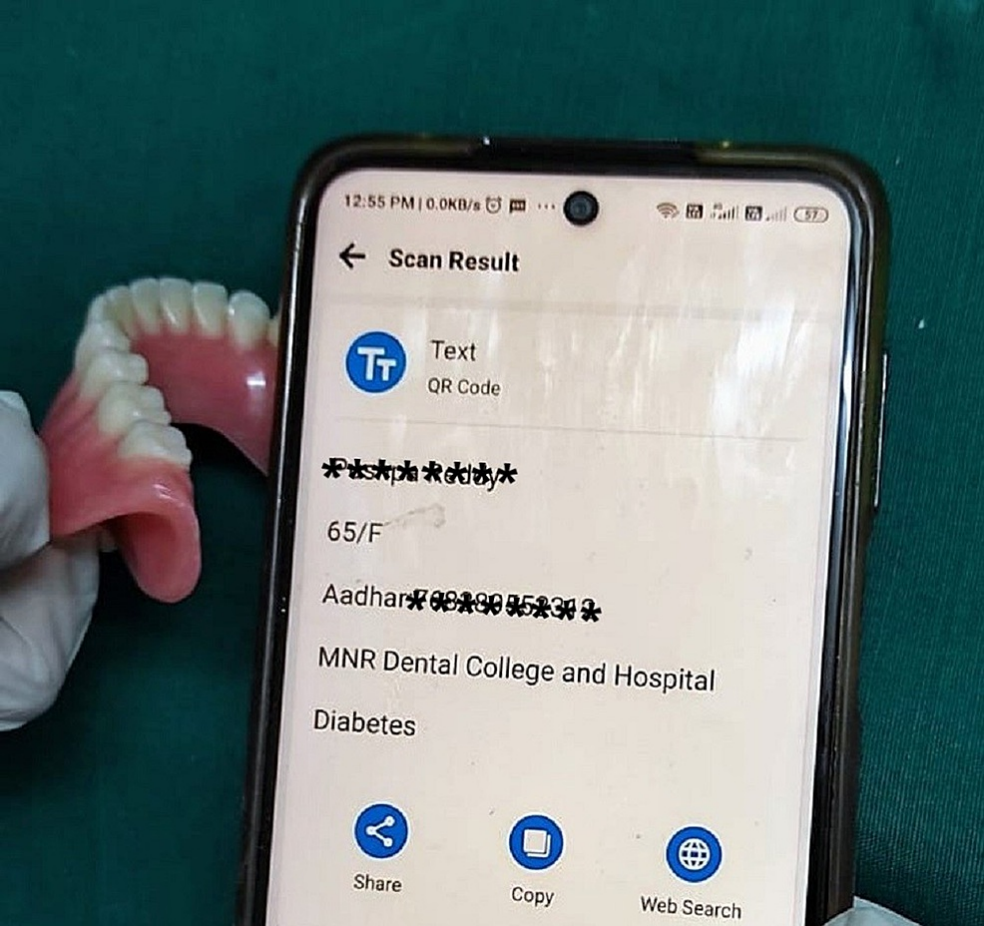
QR code translated into text. QR, quick response.

Hold a code decoder-enabled mobile camera against the denture for the code to be decoded (Figure 3). The QR code will be translated into text on the mobile phone's display (Figure 4).

## Discussion

Edentulous people have lost all or most of the key features, and it makes the identification process much more difficult. Quick identification is usually possible when a tiny identification code is embedded in the denture. The identification mark serves two important functions. First, it helps the identification of denture wearer either living or dead; and second, it helps in the identification of dentures. The former is important in cases of amnesia, unconsciousness, and postmortem, including forensic cases. The latter is important in geriatric and nursing institutions.

If lost or misplaced dentures are not labeled, it is very unlikely that they will be returned to the owner. Such a scenario will cause a lot of inconvenience and problems for the patient as well as family caregivers. It will also add to the cost and emotional stress. Hence, the importance of denture identification should be considered.

In the present article, the method to include a laser-marked stainless steel QR code stood out from the currently available denture marking methods in various ways. QR code stores a large volume of information. The dentist, already equipped with a smartphone, can use QR codes to provide rapid access to patients’ information directly within the workplace.

Laser marking is a technique utilizing a beam of light to alter the material's appearance to produce precise high-contrast markings on the surface that can be easily read or scanned. Copper vapor laser [13] has been used to produce very fine markings of a few microns to label the cobalt-chromium components of dentures. But this method stored very little information. In the suggested technique, in addition to the storage of huge data, the information can be accessed, added, or subtracted after scanning QR codes. Laser-marked QR codes on stainless steel bands can withstand high temperatures, have less chances of deterioration, and are more durable. Thermal tests revealed that metal markers are considered most ideal for postmortem identification [9].

The cost of the QR code is significantly less than the cost of using a microchip or barcode system for denture identification. In the present technique, the marker can be inserted on the lingual flange of the mandibular and buccal flange of the maxillary denture. These sites are esthetically acceptable and not removed during adjustments or routine relining procedures. It does not hinder oral function or the strength of the denture because of its small size.

Both new and existing dentures that have not been labeled can be marked using this technique. It can be also used in all metallic prostheses where denture labeling is attempted. A dentist could easily perform this procedure on their own without the help of a dental technician. The only limitation involved in laser etching is the availability of the laser engraving unit.

The simplicity of using the laser-marked stainless steel QR code can enable anyone with a smartphone to access patient information in necessary situations, thus helping to reveal the positive identity of a person when all other methods fail.

## Conclusions

The need for denture marking is important for forensic and social reasons, in case patients need to be identified individually. This article describes a simple, cheap, and durable denture marking method that can store a large amount of information. The label can be decoded with any smartphone by an on-site forensic investigator, thus allowing quick identification of the denture wearer.
